# The Potential Synergistic Modulation of AMPK by *Lippia citriodora* Compounds as a Target in Metabolic Disorders

**DOI:** 10.3390/nu11122961

**Published:** 2019-12-04

**Authors:** Mariló Olivares-Vicente, Noelia Sánchez-Marzo, José Antonio Encinar, María de la Luz Cádiz-Gurrea, Jesús Lozano-Sánchez, Antonio Segura-Carretero, David Arraez-Roman, Catherine Riva, Enrique Barrajón-Catalán, María Herranz-López, Vicente Micol

**Affiliations:** 1 Instituto de Investigación, Desarrollo e Innovación en Biotecnología Sanitaria de Elche (IDiBE), Instituto de Biología Molecular y Celular (IBMC), Miguel Hernández University (UMH), 03202 Elche, Spain; maria.olivaresv@umh.es (M.O.-V.); jant.encinar@umh.es (J.A.E.); e.barrajon@umh.es (E.B.-C.); vmicol@umh.es (V.M.); 2 Department of Analytical Chemistry, University of Granada, 18071 Granada, Spain; n.sanchez@umh.es (N.S.-M.); mluzcadiz@ugr.es (M.d.l.L.C.-G.); jesusls@ugr.es (J.L.-S.); ansegura@ugr.es (A.S.-C.); darraez@ugr.es (D.A.-R.); 3 Research and Development of Functional Food Centre (CIDAF), PTS, 18016 Granada, Spain; 4 LaPEC EA4278, Avignon University, 84000 Avignon, France; catherine.riva@univ-avignon.fr; 5 CIBER: CB12/03/30038, Fisiopatología de la Obesidad y la Nutrición, CIBERobn, Instituto de Salud Carlos III (ISCIII), 28029 Madrid, Spain

**Keywords:** *Lippia citriodora*, polyphenol, adipocyte, AMPK, molecular docking, synergy

## Abstract

*Lippia citriodora* (LC) represents a complex plant-derived source of polyphenols and iridoids that has shown beneficial properties against obesity-related metabolic disorders. The complete extract and its major compound, verbascoside, have shown AMPK-activating capacity in cell and animal models. In this work, we aimed to elucidate the contribution of the different compounds present in the LC extract on the AMPK activation capacity of the whole extract. Semipreparative reversed-phase high-performance liquid chromatography coupled to electrospray ionization time-of-flight mass spectrometry (RP-HPLC-ESI-TOF-MS) was used to identify the major compounds with bioassay-guided fractionation in an adipocyte cell model for the measurement of AMPK activity. Twenty-two compounds were identified and purified almost to homogeneity in 16 fractions, and three compounds, namely verbascoside, luteolin-7-diglucuronide and loganic acid, showed the highest AMPK-activating capacity. The synergy study using the checkerboard and fractional inhibitory concentration index (FICI) methods exhibited synergistic behavior between loganic acid and luteolin-7-diglucuronide. Molecular docking experiments revealed that these three compounds might act as direct agonists of AMPK, binding to the AMP binding sites of the gamma subunit and/or the different sites of the interaction zones between the gamma and beta subunits. Although our findings conclude that the bioactivity of the extract is mainly due to verbascoside, the synergy found between loganic acid and luteolin-7-diglucuronide deserves further research aimed to develop optimized combinations of polyphenols as a new nutritional strategy against obesity-related metabolic disorders.

## 1. Introduction

According to the World Health Organization, the global prevalence of overweight and obesity has tripled in the last four decades. In 2016, approximately 39% of the world’s adult population were overweight and approximately 13% were obese. Overweight and obesity are defined as the abnormal or excessive accumulation of fat that is closely linked to the development of chronic disorders such as cardiovascular diseases [1], type 2 diabetes [2], musculoskeletal disorders [3] and some cancers [4]. Nevertheless, obesity can be prevented by lifestyle intervention, such as restricting caloric intake and increasing regular physical activity.

For a long time, numerous plants have been used as traditional remedies against diverse metabolic disorders, including those associated with obesity [5]. In recent years, a growing number of studies have demonstrated the mechanisms by which plant compounds, especially polyphenols, confer such benefits [6,7,8,9,10,11,12,13]. Approaches such as bioguided fractionation and purification have been used to identify the compounds responsible for the health effects, but are insufficient to resolve the complexity of plant extracts. In this context, the characterization of purified compounds from plant extracts is achievable by semipreparative high-performance liquid chromatography (HPLC) coupled to reversed-phase HPLC coupled to electrospray ionization time-of-flight mass spectrometry (RP-HPLC-ESI-TOF-MS), as exemplified by our previous study which successfully identified verbascoside as the main component for the weight-lowering properties of *Lippia citriodora* (LC) [14,15,16]. Nevertheless, it has also been proposed that the presence of other minor compounds in the extract may provide synergistic effects [14]. Previous studies have shown that polyphenols from LC can ameliorate the metabolic alterations that occur in obesity by decreasing oxidative stress and mitochondrial dysfunction and modulating the expression or activity of some proteins involved in metabolic homeostasis and energy control, such as peroxisome proliferator-activated receptor alpha (PPARα) and fatty acid synthase (FASN) [14]. Evidence from cellular and animal models of obesity and from human trials suggests that the activation of AMP-activated protein kinase (AMPK) by LC polyphenols might be one of the mechanisms involved in the modulation of fat metabolism [6,13,17].

AMPK is a crucial nutrient and energy sensor that regulates energy homeostasis [18]. This protein responds to the energy demands by activating or inhibiting pathways that produce or consume ATP, respectively. AMPK consists of a heterotrimeric complex composed of one catalytic subunit (α1/α2) and two regulatory subunits (β1/β2 and γ1/γ2/γ3) [19]. The alpha subunit contains a Ser/Thr kinase domain, which comprises Thr172 within the activation loop, followed by an autoinhibitory domain and a C-terminal domain (α-CTD), which interacts with the beta subunit [20]. The beta subunit contains a carbohydrate-binding module (CBM) in its N-terminus and a C-terminal domain, which contains the αγ subunit binding sequence (αγ-SBS) domain, which is involved in binding to alpha and gamma subunits [21]. Finally, the gamma subunit contains four tandem repeats of a structural module called cystathione-β-synthase (CBS) motifs, three of which constitute adenine nucleotide binding sites [20].

AMPK is allosterically activated by AMP followed by phosphorylation of Thr172 in the alpha subunit [22]. This protein can be activated by upstream kinases such as liver kinase B1 (LKB1) and Ca^2+^/calmodulin-dependent kinase β (CaMKKβ) [23,24]. In addition, several exogenous molecules have also been described as AMPK activators, such as metformin, which has been used as an antidiabetic drug [25], or AICAR (5-aminoimidazole-4-carboxamide-1-β-4-ribofuranoside), which is a direct agonist of AMPK demonstrated by *in vitro* experiments [26]. Some plant-derived polyphenols, such as resveratrol, quercetin, and verbascoside, can also activate this protein, although the mechanism involved may vary considerably [14,27,28]. Due to their structural diversity [29], it is proposed that plant polyphenols can modulate signaling pathways leading to the activation of AMPK or even directly interact with the binding sites on the protein, facilitating enzymatic activation via phosphorylation. Computational techniques such as molecular docking and molecular dynamics are useful tools to predict the molecular interactions between the target protein and polyphenolic ligands [30]. In addition, the multitargeted character of these molecules suggests that the combinations of certain polyphenols present in plant mixtures may exert synergistic interactions [29,31,32]. Several synergy studies are available to mathematically determine the ideal proportion of these compounds in the mixtures. Nevertheless, an exhaustive and accurate design is required to obtain solid results, considering some aspects such as the biological assay and sample testing. The immunofluorescence assay is a reliable and quantitative biological test to evaluate the effect of single or combined compounds on the activity of protein targets such as AMPK [30]. In addition, checkerboard method is one of the most accepted multi-well plate designs for pairwise combinations [33]. This becomes an opportunity during the formulation of new dietary supplements focused on preventing and managing obesity-related pathologies.

Previous research has shown that an LC extract and its main component, verbascoside, have a great ability to activate AMPK in adipocytes [14]. In addition, fractions containing several polyphenols and iridoids obtained by bioassay-guided fractionation of the LC extract have exhibited the capacity to activate AMPK in hypertrophic adipocytes [34]. In this work, we aimed to determine the AMPK-activating capacity of several pure compounds present in the extract to elucidate their contribution to AMPK activation. In addition, we carried out a synergy study among the most active compounds to elucidate the existence of putative synergistic interactions. *In silico* analysis was used to predict the binding affinity of these compounds for the binding sites of AMPK. These results may contribute to the design of optimized combinations of AMPK-activating polyphenols as part of the strategy against obesity-related metabolic disorders.

## 2. Materials and Methods

### 2.1. Materials

For the characterization and semipreparative isolation of LC compounds, solvents were of HPLC-MS grade and were used as received. Acetic acid and methanol were obtained from Fluka (Sigma-Aldrich, Steinheim, Germany) and Lab-Scan (Gliwice, Poland), respectively. Water was purified with a Milli-Q system from Millipore (Bedford, MA, USA). The standard compounds loganic acid, luteolin-7-diglucuronide and isoverbascoside were purchased from PhytoLab (Vestenbergsgreuth, Germany). Verbascoside was acquired from Extrasynthese (Genay, France). The components of LC were isolated from a commercial extract of LC leaves (10% verbascoside, *w*/*w*) provided by Monteloeder, S.L. (Elche, Spain). For the evaluation of AMPK activation in adipocytes and quantitation of the compounds, an enriched LC extract (27% verbascoside, *w*/*w*) from Monteloeder was used [14]. For the AMPK activation screening and synergy study using 3T3-L1 adipocytes, the available commercial standards (i.e., loganic acid, luteolin-7-diglucuronide, verbascoside and isoverbascoside) instead of their respective purified fractions were used. The purchased compounds were dissolved in dimethyl sulfoxide (DMSO) (Sigma-Aldrich, St. Louis, MO, USA), and the isolated compounds were collected in water. Afterwards, the extract and compounds were prepared in culture medium and filtered before use in cellular experiments.

For propagation and differentiation of 3T3-L1 preadipocytes (ATCC^®^ CL-173^TM^), Dulbecco’s modified Eagle medium (DMEM) and a mix of antibiotics (penicillin/streptomycin) were acquired from Gibco (ThermoFisher Scientific, Waltham, MA, USA). Calf and fetal bovine serums were obtained from Fisher Scientific (Logan, UT, USA). 3-Isobutyl-1-methylxanthine (IBMX), dexamethasone, and insulin were obtained from Sigma-Aldrich.

### 2.2. Isolation of LC Compounds by a Semipreparative Method

For compound isolation, 50 mg of extract was dissolved in 1 mL of water, and the resulting solution was vortexed for 1 min. The sample was filtered through a 0.25 µm filter before introduction into the semipreparative HPLC system. The isolation of LC compounds was carried out using a Gilson preparative HPLC system (Gilson Inc., Middleton, WI, USA) equipped with automated liquid handling solution (model GX-271), a binary pump (model 331/332) and UV-Vis detector (model UV-Vis 156). A semipreparative Ascentis C18 column (250 × 212 mm, 10 µm) was used to separate the compounds at room temperature. The mobile phases consisted of 0.5% acetic acid in water (A) and acetonitrile (B). The multistep linear gradient developed in the previous study was employed with some changes to optimize compound purification: 0 min, 5% B; 15 min, 20% B; 21 min, 23% B; 28 min, 25% B; 32 min, 32% B; 48 min, 40% B, 65 min, 60% B; 68 min, 65% B; 70 min, 5% B; and 80 min, 5% B [34]. The flow rate used was reduced to 10 mL/min, and the injection volume was 500 µL. The UV-Vis detector was set to 240 and 280 nm, while the separated compounds were also monitored by coupling this system to the time-of-flight (TOF) mass spectrometer mentioned below.

Sixteen fractions were collected, and their solvent was evaporated at 35 °C under vacuum in a Savant SpeedVac Concentrator SC250 EXP (Thermo Scientific, Waltham, MA, USA). The residue of each fraction was dissolved in water at a concentration of 800–1033.3 ppm depending on its weight, and the samples were filtered as mentioned above before analytical characterization.

### 2.3. Analytical Characterization of the Collected Fractions by RP-HPLC-ESI-TOF-MS

Analyses of fractions were carried out in an Agilent 1200 series rapid-solution LC system (Agilent Technologies, Palo Alto, CA, USA), which included an autosampler, a binary pump and a diode-array detector (DAD). Samples (10 µL) were injected onto a Zorbax Eclipse Plus C18 column (1.8 µm, 150 × 4.6 mm) for RP-HPLC at room temperature. The flow rate was set at 0.4 mL/min, and the following multistep linear gradient was applied: 0 min, 5% B; 3 min, 10% B; 15 min, 20% B; 17 min, 23% B; 24 min, 35% B; 30 min, 40% B, 40 min, 45% B; 55 min, 60% B; 60 min, 95% B, 62 min, 5% B; and 70 min, 5% B. The initial conditions were maintained for 10 min. The elution of compounds was monitored by UV-Vis in the spectrum range of 190–950 nm by the DAD detector.

Furthermore, the HPLC system was coupled to a TOF mass spectrometer (Bruker Daltonics, Bremen, Germany) for compound identification considering an m/z range of 50–1000. This detector was equipped with an electrospray ionization (ESI) interface (model G1607A, Agilent Technologies) working in negative ion mode. The optimum values for the source and transfer parameters were described in our previous work where the external calibration was also explained [34]. The software DataAnalysis 4.0 (Bruker Daltonics) allowed the processing of accurate mass data for the molecular ions.

### 2.4. Quantitation of Compounds in the LC Extract by RP-HPLC

Stock solutions of loganic acid, luteolin-7-diglucuronide, and verbascoside were prepared in methanol:water (50:50, *v*/*v*). The first two compounds were obtained with a stock concentration of 750 µg/mL, while the verbascoside stock solution was prepared at 1500 µg/mL, taking into consideration its higher amount expected. Standard solutions were obtained by dilution from the stock solutions to final concentrations of 0.3125–500 µg/mL. For quantification of these compounds, the enriched LC extract was dissolved in methanol:water (50:50, *v*/*v*) in duplicate each at 3 and 5 mg/mL on different days (interday), and the resulting solutions were filtered using the filters mentioned above.

All samples were analyzed by RP-HPLC using the same mobile phase and multistep linear gradient described in the characterization of the collected fractions. In this case, compounds were monitored by the DAD detector at the wavelengths of 235, 320, and 350 nm.

### 2.5. Propagation and Differentiation of Preadipocytes

3T3-L1 cells were propagated in DMEM containing 10% bovine calf serum and antibiotics (100 U/mL penicillin and 100 μg/mL streptomycin) under optimal conditions (37 °C, 95% humidity, 5% CO_2_). For differentiation into adipocytes, cells were seeded in 96-well plates and maintained until confluence. Subsequently, preadipocytes were induced by incubation in DMEM containing 4.5 g/L glucose, 10% fetal bovine serum and adipogenic agents (0.5 mM IBMX, 1 μM dexamethasone and 10 μg/mL insulin) for 48 h. Then, the medium supplemented with insulin was replaced every 2–3 days until mature adipocytes were obtained. More than 90% of the cells became mature adipocytes on the 8th day of differentiation and were treated for 24 h with LC extract at 50, 100, 200 and 400 μg/mL or the identified compounds from LC at concentrations of 25, 50 and 100 μM. For the compounds dissolved in DMSO, the amount of solvent did not exceed 0.5% in cells.

### 2.6. Determination of AMPK and pAMPK Levels by Immunofluorescence Assay

Levels of activated AMPK (phospho-AMPK, pAMPK) and total AMPK were determined in adipocytes by an immunofluorescence assay. In brief, treated cells were fixed in 4% paraformaldehyde (Sigma-Aldrich), permeabilized in 0.3% Triton X-100 (Sigma-Aldrich) and blocked in 4% goat serum (Sigma-Aldrich). Then, cells were incubated with primary antibodies overnight, i.e., mouse anti-AMPK (ab80039, Abcam, Cambridge, UK) and rabbit anti-pAMPK (Thr172) (#2535, Cell Signaling Tech., Danvers, MA, USA), followed by secondary antibodies for 6 h, i.e., anti-mouse IgG-FITC (F0257) or anti-rabbit IgG-CF594 (SAB4600107), both from Sigma-Aldrich. The fluorescence signal of each protein was measured by using a cell-imaging multimode microplate reader (Cytation 3, BioTek Instruments, Winooski, VT, USA). To dismiss any cytotoxic effects, Hoechst 33342 dye (Molecular Probes, Invitrogen, Carlsbad, CA, USA) was coincubated to stain and count the nuclei by taking microphotographs with a DAPI imaging filter cube. The expression of AMPK was calculated as the ratio between total AMPK and nuclei, and the activation of AMPK was expressed as the ratio between pAMPK and total AMPK. Representative pictures of fluorescent cells were taken with Bright Field, DAPI, Texas Red and GFP filter cubes at 20×.

### 2.7. Synergy Study

Checkerboard assay was used to quantify the possible pairwise interactions between the AMPK activators. Preadipocytes were seeded and differentiated into mature adipocytes in 96-well plates, as described above. Four concentrations of each compound within the activation range of AMPK were prepared (25, 50, 100 and 200 μM). Compounds were added to the cells in pairs. For one compound, 50 μL of each preparation was added across the x-axis of the plate, while 50 μL of the other compound was added across the y-axis. Treated cells were incubated for 24 h, and AMPK activation was determined by an immunofluorescence assay. On the other hand, cell viability was assessed using Hoechst stain and none of the pairwise combinations showed cytotoxic effects (data not shown). Nontreated cells were used as a control of AMPK basal activation, and an increase in activation equal to or greater than 15% was considered statistically significant based on the AMPK activation values previously achieved for the isolated compounds. This minimum activating concentration (MAC) was used to calculate the synergistic interactions by calculating the fractional inhibitory concentration index (FICI), whose formula in activation values would be FICI = (MAC of compound X in combination/MAC of compound X alone) + (MAC of compound Y in combination/MAC of compound Y alone). It was considered a synergistic effect when FICI ≤ 0.5, an additive effect when 0.5 < FICI ≤ 1, an indifferent effect when 1 < FICI < 2, and antagonism when FICI ≥ 2, according to the interpretation of EUCAST [32,35].

### 2.8. Molecular Docking

The human AMPK (2.63 Å resolution, pdb code **5ISO**) structure was obtained from the Research Collaboratory for Structural Bioinformatics (RCSB) Protein Data Bank (PDB). The specific edition of the protein structure was made using PyMOL 2.0 software (The PyMOL Molecular Graphics System, Version 2.0 Schrödinger, LLC, at http://www.pymol.org/) without further optimization. The molecular docking experiments were performed using YASARA v19.3.26 software [36] that executed the AutoDock 4 algorithm [37], in which 999 flexible docking runs were set and clustered (7 Å) around the putative binding sites. The AMBER99 force field was used. The YASARA pH command was set to 7.4. The YASARA software calculates the Gibbs free energy variation (ΔG, kcal/mol), with more positive energy values indicating stronger binding. However, all the values included in the results of the supplementary table were included with a negative sign as the best docked. The ΔG value for the best compound docked in each cluster is shown. Dissociation constants were recalculated from the average binding energy of all compounds in each cluster. The number of docked molecules (loganic acid, luteolin-7-diglucuronide or verbascoside) included in each cluster of compounds has been indicated as “Members” in percentages in Supplementary Tables S1–S3. The theoretical dissociation constant of each ligand (loganic acid, luteolin-7-diglucuronide or verbascoside) at its putative binding site can be determined by calculating the binding energy of the ligand-receptor complex.

### 2.9. Statistical Analysis

AMPK activation values are expressed as the mean ± standard deviation. Treatments were normalized to their respective controls incubated with the amount of DMSO or water used in each treatment. Data were subjected to one-way ANOVA and Tukey’s post hoc test. Differences were considered statistically significant at *p* < 0.05. Statistical analyses were performed using GraphPad Prism version 6.00 (GraphPad Software, San Diego, CA, USA). * *p <* 0.05, ** *p <* 0.01, *** *p <* 0.001 and **** *p* < 0.0001 on the bars indicate statistically significant differences compared to the control. All experiments were performed in quintuplicate.

## 3. Results

### 3.1. Isolation of LC Compounds Using Semipreparative HPLC and Their Characterization by RP-HPLC-ESI-TOF-MS

**Figure 1:**
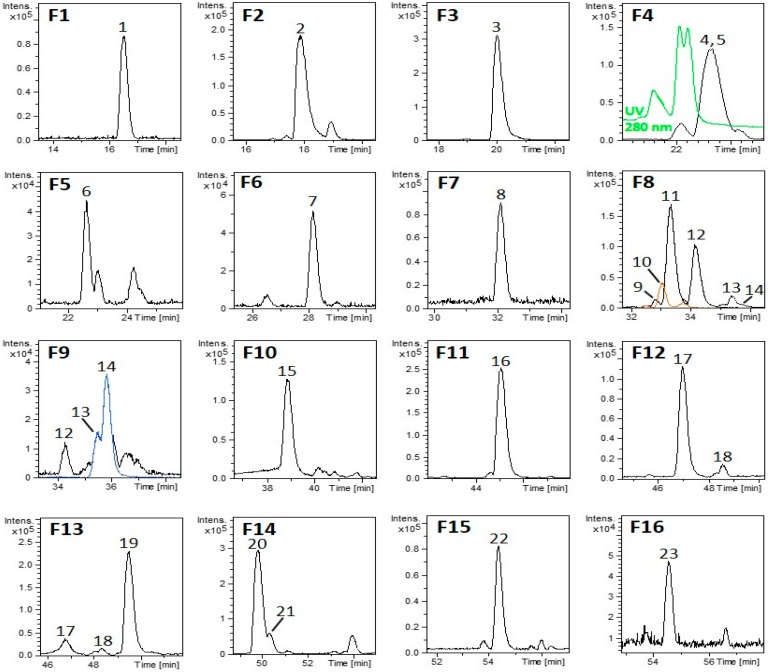
Base peak chromatograms (BPCs) of the collected fractions from the LC extract numbered as F1-F16. Peak numbers were included according to Table 1 and their elution order. The UV chromatogram at 280 nm is presented for elucidating the two isomers present in F4, while the extracted ion chromatogram (EIC) is shown in other colors for those cases that were considered necessary.

The purification semipreparative HPLC strategy was improved compared to the previous study for isolating compounds that were not commercially available [34]. A total of 16 fractions (F1-F16) were collected (Figure 1), and their composition was analyzed by the detailed RP-HPLC-ESI-TOF-MS method (Table 1). The relative percentage of each compound was estimated considering the relative area of the corresponding peak in the collected fraction.

The results showed that the main groups identified from the collected fractions were iridoids, glycosylated phenylpropanoids, flavonoids, and monoterpenoid compounds. Of the iridoids, shanziside (m/z 391) and theveside (m/z 389) were obtained completely pure in fractions F1 and F7, respectively. Fraction F2 contained mainly gardoside (m/z 373). Shanziside methyl ester (m/z 405) was obtained in fraction F6 as the major compound. Fraction F5 was composed mostly of loganic acid (m/z 375).

On the other hand, seven different fractions mainly contained glycosylated phenylpropanoids. Fraction F3 showed pure verbasoside (m/z 461), while cistanoside F isomers (m/z 487), β-hydroxy-(iso)-verbascoside (m/z 639), and martynoside (m/z 651) were present as the major compounds in F4, F9, and F16, respectively. Verbascoside (m/z 623) was isolated from fraction F11, while isoverbascoside (m/z 623) was obtained in F13. A different positional isomer of verbascoside and isoverbascoside was obtained in fraction F14: forsythoside A (m/z 623). This compound has been previously identified in the characterization of an LC extract [38]. Leucosceptoside A (m/z 637) was also present in F10 as a minor compound.

Moreover, three flavones in their glucuronide form were isolated: luteolin-7-diglucuronide (m/z 637) in F10, chrysoeriol-7-diglucuronide (m/z 651) in F12 and acacetin-7-diglucuronide (m/z 635) in F15. In this last fraction, diooflavone (m/z 621), a biflavonoid identified for the first time in LC in our previous work, was present as a minor compound [34]. Fraction F8 contained not only tuberonic acid glucoside isomers (m/z 387) but also sacranoside A (m/z 445), both monoterpenoids, and β-hydroxy-(iso)-verbascoside (m/z 639).

### 3.2. Phenylpropanoids, Iridoids, and Flavonoids from LC Modulate AMPK in Adipocytes

**Figure 2:**
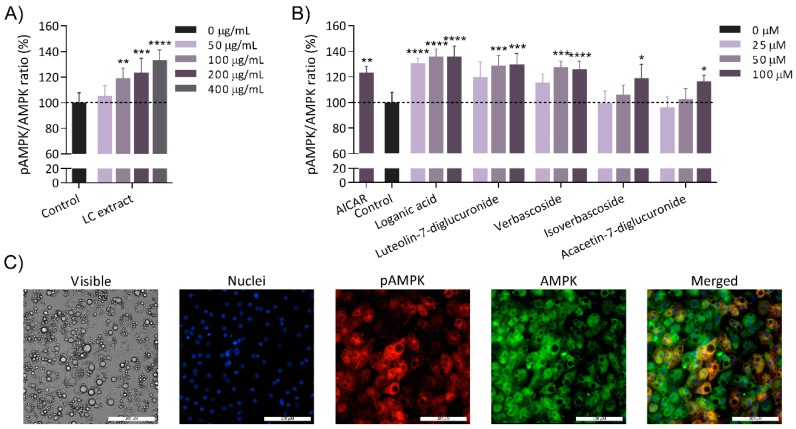
Activation of AMPK in 3T3-L1 adipocytes after 24 h of incubation with the LC extract (**A**) or the individual compounds from the LC (**B**). Protein levels of total AMPK and pAMPK were measured with an immunofluorescence assay, and the activity of AMPK was represented as the ratio between pAMPK and AMPK levels. Panel A shows the AMPK activation by the whole extract at 50, 100, 200 and 400 μg/mL. Panel B shows the AMPK activation values using the activator compounds (loganic acid, luteolin-7-diglucuronide, verbascoside, isoverbascoside and acacetin 7-diglucuronide) at 25, 50 and 100 μM. The positive control AICAR was used at 100 μM for 24 h and is represented in panel B. All the treatments are normalized to their respective controls in DMSO or water depending on the solubility of the compounds. The data are expressed as the mean ± S.D. (*n* = 5). *, **, *** and **** indicate significant differences with respect to their corresponding controls without extract/compound (*p* < 0.5, *p* < 0.01, *p* < 0.001 and *p* < 0.0001, respectively). Finally, panel (**C**) illustrates images of adipocytes in phase contrast (visible), blue fluorescence (nuclei), red fluorescence (pAMPK), green fluorescence (AMPK) and merged after the immunofluorescence assay.

Mature 3T3-L1 adipocytes were obtained to evaluate the capacity of the LC extract and its compounds to activate AMPK. After 24 h of incubation, the LC extract significantly increased the activation of AMPK through phosphorylation of Thr172 at 100, 200 and 400 μg/mL in a dose-dependent manner (Figure 2A). The level of phosphorylation of this kinase increased to 19.3% at 100 μg/mL, 23.5% at 200 μg/mL and 33.2% at 400 μg/mL extract compared to basal activation levels observed in cells incubated in the absence of the extract. The LC extract was assessed at noncytotoxic concentrations in 3T3-L1 adipocytes (Figure S1A).

For the evaluation of the isolated compounds, cells were incubated with concentrations of pure compounds equivalent to the amount of verbascoside contained in 50, 100 and 200 μg/mL LC extract (27% *w*/*w*). Accordingly, the compounds were prepared at concentrations of 25, 50 and 100 μM. Among all the compounds evaluated, five exhibited significant AMPK activation (Figure 2B). The strongest activators were loganic acid, verbascoside, and luteolin-7-diglucuronide, which increased AMPK phosphorylation up to 35.8%, 26.2% and 29.6%, respectively, with respect to the nontreated control. It is remarkable that these three compounds showed higher activation values than that exerted by the agonist AICAR at 100 μM (123.2%). In addition, the polyphenols isoverbascoside and acacetin-7-diglucuronide also activated AMPK, but this effect was weaker and only achieved at the highest concentration (119.1% and 116.5%, respectively). None of the activator compounds showed cytotoxic effects (Figure S1B).

### 3.3. Quantitation of the Main AMPK Activator Compounds in the LC Extract by RP-HPLC

In view of the results obtained above, loganic acid, luteolin-7-diglucuronide, and verbascoside were selected as putative AMPK activators in the LC extract and were quantified by RP-HPLC in the whole extract. As expected, verbascoside, the major compound of the extract comprised 27.41 ± 0.52% (*w*/*w*) of the extract, while luteolin-7-diglucuronide and loganic acid comprised 0.53 ± 0.04% and 0.05 ± 0.01% (*w*/*w*), respectively (Table 2).

### 3.4. Study of the Putative Synergistic and/or Additive Interaction between Verbascoside, Luteolin-7-Diglucuronide, and Loganic Acid Derived from LC on AMPK Activation

**Figure 3:**
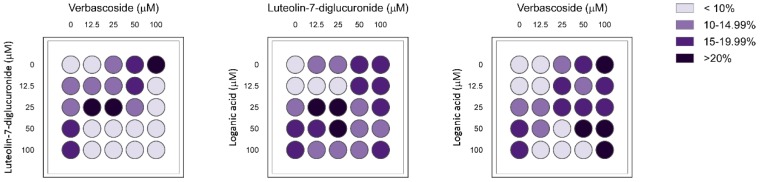
Scheme of the checkerboard method for FICI determination of the pairwise combinations between the best AMPK activators derived from the LC extract. Four concentrations of the selected compounds from LC (loganic acid, luteolin-7-diglucuronide, and verbascoside) were added to adipocytes in 96-well plates in pairwise combinations. One compound was added on the x-axis across the plate at the indicated concentrations, and another compound was added along the y-axis. Treated adipocytes were incubated for 24 h, and AMPK values were obtained by an immunofluorescence assay. An increase in AMPK activation of 15% or greater with respect to nontreated cells (0 μM) was considered significant (*n* = 2).

To explore the possible synergistic effects on AMPK modulation, we performed a pharmacological synergy study with the three most active compounds of LC (loganic acid, luteolin-7-diglucuronide, and verbascoside). To address this, the compounds were added to the adipocytes in paired combinations at different concentrations using the checkerboard method. After 24 h of incubation, AMPK activation was quantified by an immunofluorescence assay to calculate the FICI values of each combination (Figure 3). The FICI values of the three possible combinations between loganic acid, verbascoside, and luteolin-7-diglucuronide are represented in Table 3. The FICI analyses showed a synergistic effect for the combination of loganic acid + luteolin-7-diglucuronide, with a FICI value = 0.5. The combinations loganic acid + verbascoside and verbascoside + luteolin-7-diglucuronide showed FICI values = 0.75, indicating an additive effect in both cases.

### 3.5. A Virtual Screening Approach Shows the Potential Direct Interaction of Verbascoside, Luteolin-7-Diglucuronide, and Loganic Acid with AMPK

Molecular docking techniques aim to predict the structure of the ligand-receptor complex using computational methods and need high-resolution structural information on the target structure. The docking software has a scoring function to make an approximate calculation of the binding free energy between the target (human AMPK) and the ligand (loganic acid, luteolin-7-diglucuronide or verbascoside) in each binding pose. To search for potential binding sites in each protein of interest, from the aforementioned compounds, a global molecular docking procedure was performed with AutoDock 4 [37] implemented in YASARA software [39].

**Figure 4:**
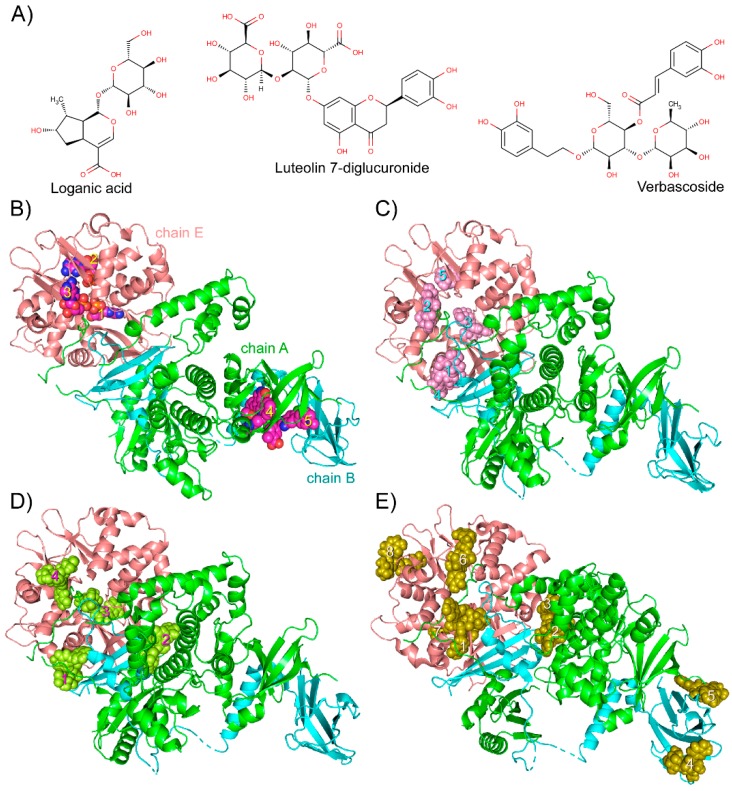
Illustration of the secondary structure for human AMPK with docked loganic acid (**C**), luteolin-7-diglucuronide (**D**) and verbascoside (**E**). For each cluster of the docked compound (loganic acid carbon atoms in pink color, luteolin-7-diglucuronide carbon atoms in lemon color and verbascoside carbon atoms in olive color), the molecules (spheres) with better binding energies are shown (a number indicates each cluster of different compounds, see Supplementary Tables). The three AMPK subunits have been shown with different colors (alpha-2 in green as *chain A*, beta-1 in blue as *chain B*, and gamma-1 in salmon as *chain E*). The number of each cluster is indicated. The 5ISO structure was used (panels (**B**), (**C**), (**D**), and (**E**)), and panel B shows the three AMP binding sites (1, 2, 3), the ATP binding site in the catalytic domain (4), and the regulatory site in the interaction zone between the alpha-2 and beta-1 subunits (5). Finally, panel (**A**) shows the molecular structures of loganic acid, luteolin-7-diglucuronide, and verbascoside. The figure was prepared using PyMOL 2.0 software.

The results obtained are shown in Figure 4 and Supplementary Tables S1–S3. A first observation of the results shows that for the three all ligands, the clusters with lower ΔG values for each compound are in the regulatory positions previously described in the literature. Figure 4B shows the 3 AMP binding sites in the gamma subunit, the ATP binding site in the catalytic subunit and the regulatory site at the interface of the alpha and beta subunits (see chain E, A, and B in Figure 4B, respectively; ligands are represented as spheres). None of the three compounds tested were located at the ATP binding site, while various occupancies of any of the three AMP binding sites can be observed in the gamma subunit. This would explain their behavior as AMPK agonists. A second interesting observation is that none of the three compounds localized clusters with high affinity in the binding site that is established in the zone of interaction between the AMPKα catalytic and AMPKβ regulatory subunits. Finally, a third observation of interest is that different sites of high affinity have been found in the interaction zones between the gamma and beta subunits for the three compounds analyzed (Figure 4 and Supplementary Tables S1–S3). Occupation of these sites could increase the catalytic activity of the alpha subunit.

## 4. Discussion

Previous studies have highlighted the putative existence of a synergistic pharmacological effect among several polyphenols present in the LC extract to ameliorate lipid metabolism through AMPK activation [14]. To explore this, we have isolated the major compounds from the extract, identified those that are responsible for the AMPK-activating capacity in a cellular model and performed a synergy study using the most active ones in pair combinations.

In a previous work, we performed fractionation of an LC extract by semipreparative chromatography to obtain fractions with a lower possible number of compounds [34]. Nevertheless, these fractions contained a few compounds, and the evaluation of these fractions on hypertrophic 3T3-L1 adipocytes revealed that most of the fractions exerted the capacity to activate AMPK, probably due to the existence of active compounds in several fractions. In this work, due to the optimization of the semipreparative HPLC purification, we achieved the isolation of the components of LC to homogeneity and we identified the candidates responsible for AMPK activity in an adipocyte model. For this purpose, we optimized the semipreparative purification process to improve chromatographic separation and to obtain nearly pure compounds in each fraction [40].

A total of 22 compounds contained in 16 fractions were identified in the LC extract. Among them, five iridoids (shanziside, gardoside, loganic acid, shanziside methyl ester and theveside), nine glycosylated phenylpropanoids (verbasoside, two cistanoside F isomers, β-hydroxy-(iso)-verbascoside, verbascoside, isoverbascoside, leucosceptoside A and martynoside), four flavones (luteolin-7-diglucuronide, chrysoeriol-7-diglucuronide, diooflavone and acacetin-7-diglucuronide) and three monoterpenoids (tuberonic acid glucoside isomers and sacranoside A) were detected, which is in agreement with previous work [34]. Additionally, the compound forsythoside A, which had been previously identified in this plant [38], was also found in the extract.

In contrast to a previous work [34], most of the identified LC compounds were obtained in fractions with high purity, such as shanziside in F1, gardoside in F2, verbasoside in F3, shanziside methyl ester in F6, theveside in F7, luteolin-7-diglucuronide in F10, and acacetin-7-diglucuronide in F15. Moreover, the isolation of verbascoside in F11 and martynoside in F16 was also achieved in a previous study. Additionally, other fractions contained at most two or three compounds, with one compound being the most abundant (cistanoside F isomers in F4, loganic acid in F5, tuberonic acid glucoside isomer in F8, β-hydroxy-(iso)-verbascoside in F9, chrysoeriol-7-diglucuronide in F12, isoverbascoside in F13 and forsythoside A in F14) together with a few minor compounds.

We tested the selected LC pure compounds, either from the purified fractions or from a commercial source, in mature 3T3-L1 adipocytes to evaluate the AMPK activation capacity. Among all the compounds, loganic acid, luteolin-7-diglucuronide, and verbascoside showed the strongest capacity to activate AMPK, whereas isoverbascoside and acacetin-7-diglucuronide exhibited a weak effect on AMPK activation at the highest concentration evaluated (100 μM). In agreement with our previous results [34], the five activator compounds evaluated here in pure form were also present in the LC fractions that exhibited AMPK-activating capacity in hypertrophic adipocytes. Our results suggest that these compounds could be responsible for the activity of these fractions and therefore account for the activity of the whole extract. In the preceding study, other fractions that showed AMPK-activating capacity contained compounds that did not exhibit AMPK activity in our study. Nevertheless, a reliable comparison is difficult to establish because the concentrations tested and incubation time for the previous study were higher than those selected for the present study [34].

In our results, similar levels of AMPK activation were achieved with 200 μg/mL LC extract (27% verbascoside) and with 50 and 100 µM pure verbascoside. As verbascoside is the major compound of the extract, we conclude that this phenylpropanoid is one of the main factors responsible for the AMPK-activating capacity of the extract. In agreement with these findings, bioassay-guided fractionation of an olive leaf extract showed that those fractions mainly composed of verbascoside exhibited the highest capacity to activate AMPK in adipocytes [30]. In addition, the capacity of pure verbascoside to regulate energy pathways in hypertrophic adipocytes was also previously demonstrated [14] through the mRNA expression up-regulation of PPARα and the mRNA expression down-regulation of FASN, which are related to lipid and energy homeostasis. However, the activity of pure verbascoside was lower than that obtained by the whole extract at equivalent verbascoside concentrations, suggesting the presence of other active compounds contributing to this effect. Accordingly, we carried out a synergy study with the most active compounds of LC (loganic acid, luteolin-7-diglucuronide, and verbascoside) to determine whether these compounds, in their appropriate proportions, could enhance the AMPK activation capacity. Isoverbascoside and acacetin-7-diglucuronide were discarded in this analysis because they showed weak AMPK activation values in the cellular assay, and this activation was achieved at concentrations difficult to achieve *in vivo*.

We evaluated the three most active compounds for their capacity to present pharmacological interactions in the activation of AMPK in pairwise combinations. The combinations of verbascoside + luteolin-7-diglucuronide and verbascoside + loganic acid showed additive effects on AMPK activation, but a synergistic interaction between loganic acid and luteolin-7-diglucuronide was found. Nevertheless, verbascoside represented 27.41 ± 0.52% (*w*/*w*) of the extract, whereas loganic acid and luteolin-7-diglucuronide constituted 0.05 ± 0.01 and 0.53 ± 0.01% (*w*/*w*), respectively. Therefore, a concentration of LC extract of 200 μg/mL in the adipocyte assay contained concentrations of 87.8 μM verbascoside, 1.7 μM luteolin-7-diglucuronide and 0.27 μM loganic acid. The low relative proportions of these two last compounds in the extract led us to conclude that the AMPK-activating capacity of LC was mainly due to verbascoside. Despite this, our data suggest that the presence of these minor compounds used in adequate proportions, together with verbascoside, could synergistically enhance the bioactivity of properly designed combinations of polyphenols.

It should be considered that the *in vivo* activity of an LC extract depends essentially on the absorption and metabolism of its compounds in the gut, so that the effector metabolites in the target tissue may differ considerably from the ingested compounds. Previously, it was reported that certain phenolic metabolites of LC in plasma samples of rats after oral administration of an extract correlated with antioxidant protection in blood cells [10]. In that study, verbascoside was found to be the major metabolite, suggesting that this phenylpropanoid can be absorbed in its native form. In addition, low levels of luteolin-7-diglucuronide were also detected in plasma, probably by a prior deglycosylation of this and other forms of the flavone in the gut barrier and subsequent glucuronidation, as reported from other plant sources [41].

With the aforementioned, it is reasonable to postulate that verbascoside and luteolin-7-diglucuronide may reach molecular targets *in vivo*. Regarding loganic acid, this iridoid has been proposed to exert antiobesity effects in an ovariectomized female mouse model and reduce adipogenesis in 3T3-L1 preadipocytes through regulation of some adipogenesis-related genes [42]. Nevertheless, whether this compound is absorbed in its native form or undergoes phase II biotransformation reactions deserves further research.

Furthermore, our computational docking data suggest that the three polyphenols studied could potentially act directly as AMPK agonists. As derived from the molecular docking experiment, the three most active compounds of LC showed poses with ΔG values below -9 kcal/mol for several regulatory positions of AMPK. Some of these positions corresponded to one of the three AMP binding sites of the gamma subunit, suggesting that the LC compounds may activate AMPK by mimicking the allosteric mechanism of AMP. This mechanism has been previously described for other agonists, such as AICAR, which is phosphorylated inside the cell into AICA-riboside monophosphate (ZMP), an analog of AMP [26]. Similarly, the occupation of LC compounds in different sites of the interaction zones between the gamma and beta subunits could also exert an increase in the catalytic activity of AMPK. Finally, none of the three LC compounds seemed to interact with either the ATP binding site of the alpha subunit or the interface established between the alpha subunit kinase domain (KD) and the beta subunit CBM. The latter is called the allosteric drug and metabolite (ADaM) site, as it has been described as a binding site of activators such as A-769662 [43], compound 911 and salicylate [44,45]. Therefore, the additive and synergistic effects between these three LC bioactive compounds on AMPK activity could be explained through the cointeraction of these compounds in different binding sites of the protein. Although we show here that some LC compounds may be direct activators of AMPK, the possibility that these compounds could target other upstream kinases of AMPK should also be further studied.

In agreement with our findings, several studies in humans and in animal models with botanical extracts or ingredients containing in part or fully verbascoside have shown significant cardiometabolic benefits. Recently, a dietary supplement containing verbascoside and anthocyanins have exhibited vascular and metabolic benefits in a randomized controlled trial with overweight and obese subjects [6,13]. Moreover, the consumption of low doses of an olive leaf extract containing verbascoside, secoiridoids and flavonoids during 5 weeks in high fat diet (HFD)-induced obese mice prevented the development of obesity, glucose intolerance, insulin resistance and endothelial dysfunction [46]. Last, the long-term intake of verbascoside 100 mg significantly reduced platelet aggregation values in patients with cardiovascular risk factors [47]. However, as shown in the present study, verbascoside is not the only active compound present in the LC extract. Therefore, the contribution of other active compounds, such as loganic acid and luteolin-7-diglucuronide, in cardiovascular amelioration deserves further research.

## 5. Conclusions

Previous research has noted that equivalent concentrations of verbascoside in an LC extract exerted a higher capacity to regulate lipid homeostasis through AMPK-dependent mechanisms than pure verbascoside in cell models, suggesting a putative synergistic pharmacological interaction between verbascoside and other components of the extract. In this study, the compounds derived from the LC extract were purified almost to homogeneity by semipreparative high-resolution HPLC-MS, and their capacity for activation was determined in an adipocyte cell model. Our results support that although the most active compounds in the AMPK-activating assay were loganic acid, luteolin-7-diglucuronide, and verbascoside, the concentrations of loganic acid and luteolin-7-diglucuronide in the extract were too low to contribute significantly to the AMPK-activating capacity of the extract. However, the putative synergistic behavior found for the loganic acid + luteolin-7-diglucuronide combination should be further studied in cell or animal models to determine the capacity of tailor-made combinations of these three polyphenols against obesity-induced metabolic stress. Since verbascoside and luteolin-7-diglucuronide have been previously detected in plasma samples in animal models, we conclude that these polyphenols may contribute to the beneficial effects observed *in vivo*. Moreover, our molecular docking results suggest that the increase in the catalytic activity of AMPK could occur through their direct interaction with the AMP binding sites on the gamma subunit or other interaction zones between the gamma and beta subunits.

## Figures and Tables

**Table 1:** Retention time (RT) and mass spectral data of the compounds characterized in the collected fractions of LC by RP-HPLC-ESI-TOF-MS in negative mode.

Peak	RT (min)	[M − H]^−^ Measured	[M − H]^−^ Calculated	Error (ppm)	mSigma	Molecular Formula	Proposed Compound	Fraction	Relative Area (%) in Fraction
1	16.31	391.1236	391.1246	4.1	6.6	C 16 H 24 O11	Shanziside	F1 *	100.0
2	17.79	373.1131	373.114	2.4	2.7	C 16 H 22 O 10	Gardoside	F2 *	91.2
3	20.03	461.165	461.1664	3.2	1.5	C 20 H 30 O 12	Verbasoside	F3 *	100.0
4	22.5	487.1474	487.1457	−3.5	1.6	C 21 H 28 O 13	Cistanoside F isomer	F4 *	38.8
5	22.67	487.148	487.1457	−4.6	2.7	C 21 H 28 O 13	Cistanoside F isomer	F4 *	52.1
6	22.81	375.1238	375.1297	15.8	9.8	C 16 H 24 O 10	Loganic acid	F5 *	57.1
7	28.27	405.1377	405.1402	6.4	11.6	C 17 H 26 O 11	Shanziside methyl ester	F6 *	90.9
8	32.07	389.1082	389.1089	1.9	1.1	C 16 H 22 O 11	Theveside	F7 *	100.0
9	32.82	387.1657	387.1661	0.9	4.6	C18 H27 O9	Tuberonic acid glucoside isomer	F8	2.1
10	32.97	445.2075	445.2079	1	5.6	C 21 H 34 O 10	Sacranoside A	F8	7.1
11	33.37	387.1651	387.1661	2.5	6.9	C 18 H 28 O 9	Tuberonic acid glucoside isomer	F8 *	51.8
12	34.13	387.2024	387.2024	0.1	3.6	C 19 H 32 O8	UK	F8 */F9	32.4/12.1
13	35.38	639.1914	639.1931	2.6	23.7	C 29 H 36 O 16	β-hydroxy-(iso)-verbascoside	F8/F9 *	5.1/18.3
14	35.95	639.1909	639.1931	3.3	36	C 29 H 36 O 16	β-hydroxy-(iso)-verbascoside	F8/F9 *	1.4/51.6
15	38.83	637.1024	637.1046	3.6	4	C 27 H 26 O 18	Luteolin-7-diglucuronide	F10 *	87.9
16	45.05	623.1936	623.1981	7.3	3.3	C 29 H 36 O 15	Verbascoside	F11 *	84.4
17	47.04	651.1229	651.1203	−4	2.3	C 28 H 28 O 18	Chrysoeriol-7-diglucuronide	F12 */F13	90.9/3.1
18	48.55	621.1879	621.1766	−18.2	21.8	C 36 H 30 O 10	Diooflavone	F12/F13	6.9/3.2
19	49.17	623.2108	623.1981	13.5	8.8	C 29 H 36 O 15	Isoverbascoside	F13 *	80.1
20	49.87	623.1992	623.1981	−1.9	0.2	C 29 H 36 O 15	Forsythoside A	F14 *	78.2
21	50.44	637.2126	637.2138	1.9	11.3	C 30 H 38 O 15	Leucosceptoside A	F14	9.7
22	54.42	635.1277	635.1254	−3.7	3.4	C 28 H 28 O 17	Acacetin-7-diglucuronide	F15 *	90.2
23	54.63	651.2299	651.2294	−0.7	3.8	C 31 H 40 O 15	Martynoside	F16 *	89.4

* indicates the major contribution of a compound in the remarked fraction.

**Table 2:** Quantitation of the AMPK-activating compounds in the LC extract by RP-HPLC and analytical parameters from the standard curve used for the quantitation.

Compound	RT (min)	LOD (μg/mL)	LOQ (μg/mL)	Linear Range (μg/mL)	Linear Regression Equation	*r* ^2^	% (*w*/*w*) of Compoundin the LC Extract
Loganic acid	14.075	0.15625	0.3125	0.3125–750	y = 19.035x + 60.018	0.9986	0.05 ± 0.01
Luteolin-7-diglucuronide	22.728	0.15625	0.3125	0.3125–750	y = 18.543x − 103.53	0.9996	0.53 ± 0.01
Verbascoside	31.066	0.15625	0.3125	0.3125–1500	y = 11.543x − 40.687	0.9996	27.41 ± 0.52

Compounds were monitored by the DAD detector at 235, 320, and 350 nm wavelengths for loganic acid, verbascoside, and luteolin-7-diglucuronide, respectively.

**Table 3:** FICI values and synergy interpretation for the pairwise combinations between the best AMPK activators.

Combination	FICI	Effect
Verbascoside + Luteolin-7-diglucuronide	0.75	Additivity
Luteolin-7-diglucuronide + Loganic acid	0.5	Synergy
Verbascoside + Loganic acid	0.75	Additivity

FICI values were calculated according to the formula: FICI = (MAC of compound X in combination/MAC of compound X alone) + (MAC of compound Y in combination/MAC of compound Y alone).

## Supplementary Materials

The following are available online at https://www.mdpi.com/2072-6643/11/12/2961/s1, Figure S1: Cell viability of adipocytes 3T3-L1 after 24 h of incubation with LC extract (**A**) or individual compounds (**B**). Number of cells was counted by staining nuclei with Hoechst. AICAR was incubated at 100 μM for 24 h and represented in panels (**B**). All the treatments are normalized to their respective controls in DMSO or water depending on the solubility of the compounds. The data are expressed as the mean ± S.D (*n* = 5), Table S1: Details of the interaction of loganic acid docked to the human AMPK (see Figure 4C), Table S2: Details of the interaction of luteolin 7-diglucuronide docked to the human AMPK (see Figure 4D), Table S3: Details of the interaction of verbascoside docked to the human AMPK (see Figure 4E).

## Abbreviations

AICAR	5-Aminoimidazole-4-carboxamide-1-β-4-ribofuranoside
AMP	adenosine 5′-monophosphate
AMPK	AMP-activated protein kinase
CBM	carbohydrate-binding module
ΔG	Gibbs free energy variation
FASN	fatty acid synthase
LC	Lippia citriodora
MAC	Minimum activating concentration
pAMPK	phospho-AMPK
PPARα	peroxisome proliferator-activated receptor alpha
RP-HPLC-ESI-TOF-MS	reversed-phase high-performance liquid chromatography coupled to electrospray ionization time-of-flight mass spectrometry
