# Supplementary material for: The Potential Synergistic Modulation of AMPK by Lippia citriodora Compounds as a Target in Metabolic Disorders
Source: Nutrients. 2019 Dec 4;11(12):2961. doi: 10.3390/nu11122961 (PMC6950112; doi:10.3390/nu11122961)
Supplement: Supplementary file 1 [file nutrients-11-02961-s001.pdf]

Supplementary

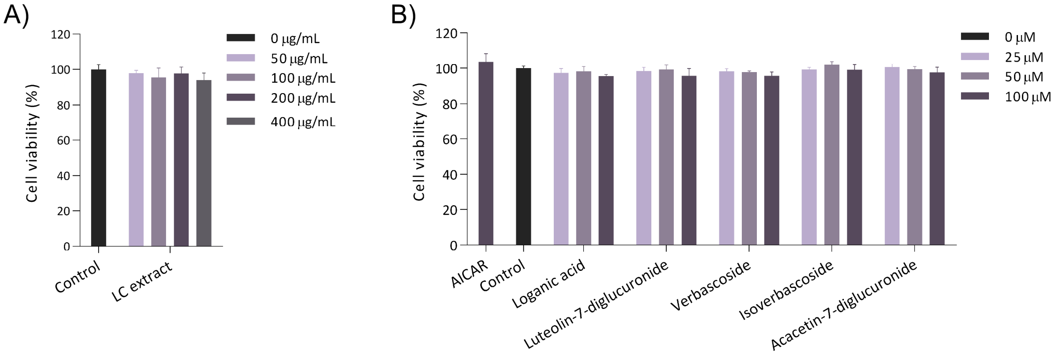

Supplementary Figure 1. Cell viability of adipocytes 3T3-L1 after 24 h of incubation with LC extract (A) or individual compounds (B). Number of cells was counted by staining nuclei with Hoechst. AICAR was incubated at 100 μM for 24 h and represented in panels **B**. All the treatments are normalized to their respective controls in DMSO or water depending on the solubility of the compounds. The data are expressed as the mean ± S.D (n = 5).

Supplementary Table 1. Details of the interaction of loganic acid docked to the human AMPK (see **Figure 4C**).

Cluster number	ΔG, [kcal/mol]	Dissoc. constant, [μM]	Members	Residues of the AMPK that contact loganic acid
1	-9.735±1.015	0.073095	4.85%	ARG(A)-369, LYS(A)-399, ALA(A)-400, TRP(A)-402, ILE(B)-214, LEU(B)-215, ASN(B)-216, LYS(B)-217, ASP(B)-218, THR(B)-219, LEU(B)-228, LEU(B)-242, LEU(B)-265, TYR(B)-267, LYS(B)-47, THR(B)-66, ASN(B)-67
2	-9.626±0.899	0.087846	6.06%	LYS(A)-364, PRO(A)-365, HIS(A)-366, PRO(A)-367, ARG(A)-369, MET(A)-370, PRO(A)-371, ILE(E)-240, PHE(E)-244, ASP(E)-245, ASN(E)-248, LEU(E)-266, HIS(E)-268, ARG(E)-269, TYR(E)-272, PHE(E)-273, GLU(E)-274, GLY(E)-275, VAL(E)-276, LEU(E)-277, VAL(E)-297
3	-9.603±0.892	0.091275	2.12%	ARG(E)-70, LEU(E)-74, GLY(E)-84, MET(E)-85, LEU(E)-86, THR(E)-87, THR(E)-89, ASP(E)-90, ARG(E)-118, TYR(E)-121, LYS(E)-127, LEU(E)-129, VAL(E)-130, CYS(E)-131, LYS(E)-149, ILE(E)-150, HIS(E)-151, ARG(E)-152, LEU(E)-153, PRO(E)-154, LYS(E)-243
4	-9.340±1.363	0.142345	3.83%	LEU(A)-373, LYS(A)-398, LYS(A)-399, ALA(A)-400, TRP(A)-402, LEU(B)-215, LEU(B)-242, LYS(B)-245, ASP(B)-246, VAL(B)-248, VAL(B)-250, LEU(B)-265, TYR(B)-267, LYS(E)-59, PHE(E)-62, ALA(E)-63, THR(E)-66, ASN(E)-67

5	-9.121±1.164	0.206011	2.02%	GLY(E)-199, THR(E)-200, ALA(E)-202, ASN(E)-203, ILE(E)-204, ALA(E)-205, HIS(E)-223, ARG(E)-224, VAL(E)-225, SER(E)-226, ALA(E)-227, LEU(E)-228, PRO(E)-229, ARG(E)-299, ILE(E)-312, VAL(E)-313, SER(E)-314, SER(E)-316, ASP(E)-317

Supplementary Table 2. Details of the interaction of luteolin 7-diglucuronide docked to the human AMPK (see **Figure 4D**).

Cluster number	ΔG, [kcal/mol]	Dissoc. constant, [μM]	Members	Residues of the AMPK that contact luteolin 7-diglucuronide
1	-11.696±1.722	0.002669	1.11%	LEU(A)-373, VAL(A)-397, LYS(A)-398, LYS(A)-399, ALA(A)-400, LYS(A)-401, TRP(A)-402, LEU(B)-215, ASN(B)-216, LEU(B)-242, SER(B)-243, LYS(B)-245, ASP(B)-246, VAL(B)-248, VAL(B)-250, TYR(B)-267, LYS(E)-47, LYS(E)-59, PHE(E)-62, ALA(E)-63, THR(E)-66, ASN(E)-67
2	-10.279±1.223	0.029203	1.01%	ARG(A)-369, MET(A)-370, PRO(A)-371, PRO(A)-372, LEU(A)-373, VAL(A)-397, LYS(A)-398, LYS(A)-399, ALA(A)-400, TRP(A)-402, LEU(B)-215, LYS(B)-217, ASP(B)-218, THR(B)-219, ILE(B)-221, LEU(B)-242, LEU(B)-265, LYS(E)-47, VAL(E)-65, THR(E)-66, ASN(E)-67, GLY(E)-68
3	-11.485±1.304	0.00381	0.91%	ARG(E)-70, GLY(E)-84, MET(E)-85, LEU(E)-86, THR(E)-87, ILE(E)-88, THR(E)-89, ASP(E)-90, ASN(E)-93, TYR(E)-121, LYS(E)-127, PRO(E)-128, LEU(E)-129, VAL(E)-130, ASN(E)-148, LYS(E)-149, ILE(E)-150, HIS(E)-151, ARG(E)-152, PHE(E)-220, VAL(E)-221, ARG(E)-224, SER(E)-226, LYS(E)-243
4	10.427±1.529	0.022743	2.12%	LYS(A)-364, PRO(A)-365, HIS(A)-366, PRO(A)-367, GLU(A)-368, ARG(A)-369, MET(A)-370, PRO(A)-371, PHE(E)-244, ASP(E)-245, HIS(E)-268, ARG(E)-269, HIS(E)-271, TYR(E)-272, PHE(E)-273, GLU(E)-274, GLY(E)-275, LEU(E)-277

Supplementary Table 3. Details of the interaction of verbascoside docked to the human AMPK (see **Figure 4E**).

Cluster number	ΔG, [kcal/mol]	Dissoc. constant, [μM]	Members	Residues of the AMPK that contact verbascoside
1	-10.500±1.226	0.020111	1.05%	ARG(A)-369, MET(A)-370, PRO(A)-371, PRO(A)-372, LEU(A)-373, LYS(A)-399, ALA(A)-400, TRP(A)-402, LYS(B)-217, ASP(B)-218, THR(B)-219, PRO(B)-225, LEU(B)-228, LEU(B)-242, VAL(B)-250, LEU(B)-265, TYR(B)-267, LYS(E)-47,

				VAL(E)-65, THR(E)-66, ASN(E)-67, GLY(E)-68
2	-10.190±0.440	0.033937	2.50%	ASP(A)-128, HIS(A)-131, ARG(A)-132, MET(A)-164, ALA(A)-191, PRO(A)-193, GLU(A)-194, ILE(A)-197, THR(A)-258, ILE(A)-259, LYS(A)-260, GLU(A)-291, HIS(B)-209, GLU(B)-230, PRO(B)-231, ASN(B)-232, HIS(B)-233, LYS(B)-259, TYR(B)-261, THR(E)-44
3	-10.120±1.228	0.038193	3.61%	GLU(A)-295, GLU(A)-298, LYS(A)-299, PHE(A)-300, GLU(A)-301, ILE(A)-327, ASN(A)-330, ARG(A)-331, MET(A)-334, LYS(B)-258, SER(E)-35, HIS(E)-36, ARG(E)-37, TYR(E)-39, ASP(E)-40, ASN(E)-135
4	-9.700±0.540	0.077597	4.21%	TRP(B)-84, GLY(B)-86, GLY(B)-87, GLY(B)-88, LYS(B)-89, LYS(B)-126, VAL(B)-129, ASP(B)-130, GLN(B)-132, TRP(B)-133, THR(B)-134, HIS(B)-135, ASP(B)-136, PRO(B)-137, GLY(B)-147, THR(B)-148, VAL(B)-149
5	-9.540±0.362	0.101655	3.85%	GLY(A)-9, ARG(A)-10, VAL(A)-11, LYS(A)-12, LEU(B)-103, PRO(B)-104, LEU(B)-105, THR(B)-106, ARG(B)-107, VAL(B)-113, ALA(B)-114, ILE(B)-115, LEU(B)-116, ASP(B)-117
6	-11.777±1.159	0.002328	1.07%	LYS(A)-364, PRO(A)-365, HIS(A)-366, PRO(A)-367, GLU(A)-368, ARG(A)-369, MET(A)-370, PRO(A)-371, ILE(E)-240, PHE(E)-244, ASP(E)-245, HIS(E)-268, ARG(E)-269, TYR(E)-272, PHE(E)-273, GLU(E)-274, GLY(E)-275, VAL(E)-276, LEU(E)-277, LYS(E)-278, ARG(E)-291, ALA(E)-295, VAL(E)-297, ARG(E)-299
7	-10.240±0.232	0.031190	1.06%	VAL(E)-83, GLY(E)-84, MET(E)-85, THR(E)-89, ASN(E)-93, ARG(E)-118, TYR(E)-121, LEU(E)-122, GLN(E)-123, ASP(E)-124, SER(E)-125, LYS(E)-127, PRO(E)-128, LEU(E)-129, VAL(E)-130, ASN(E)-148, LYS(E)-149, ILE(E)-150, ARG(E)-152, PRO(E)-154, ARG(E)-224, LYS(E)-243
8	-9.880±0.142	0.057267	2.03%	ILE(E)-204, ALA(E)-205, MET(E)-206, VAL(E)-207, ARG(E)-208, THR(E)-209, THR(E)-210, THR(E)-211, PRO(E)-212, VAL(E)-215, ILE(E)-219, VAL(E)-231, ASP(E)-232, GLU(E)-233
